# Genome wide association analysis for yield related traits in maize

**DOI:** 10.1186/s12870-022-03812-5

**Published:** 2022-09-21

**Authors:** Tingru Zeng, Zhaodong Meng, Runqing Yue, Shouping Lu, Wenlan Li, Wencai Li, Hong Meng, Qi Sun

**Affiliations:** Maize Institute, Shandong Academy of Agricultural Sciences, Jinan, China

**Keywords:** Maize, Yield related trait, Genome-wide association study, Quantitative trait nucleotide, Marker-assisted selection

## Abstract

**Background:**

Understanding the genetic basis of yield related traits contributes to the improvement of grain yield in maize.

**Results:**

Using 291 excellent maize inbred lines as materials, six yield related traits of maize, including grain yield per plant (GYP), grain length (GL), grain width (GW), kernel number per row (KNR), 100 kernel weight (HKW) and tassel branch number (TBN) were investigated in Jinan, in 2017, 2018 and 2019. The average values of three environments were taken as the phenotypic data of yield related traits, and they were statistically analyzed. Based on 38,683 high-quality SNP markers in the whole genome of the association panel, the MLM with PCA model was used for genome-wide association analysis (GWAS) to obtain 59 significantly associated SNP sites. Moreover, 59 significantly associated SNPs (*P* < 0.0001) referring to GYP, GL, GW, KNR, HKW and TBN, of which 14 SNPs located in yield related QTLs/QTNs previously reported. A total of 66 candidate genes were identified based on the 59 significantly associated SNPs, of which 58 had functional annotation.

**Conclusions:**

Using genome-wide association analysis strategy to identify genetic loci related to maize yield, a total of 59 significantly associated SNP were detected. Those results aid in our understanding of the genetic architecture of maize yield and provide useful SNPs for genetic improvement of maize.

**Supplementary Information:**

The online version contains supplementary material available at 10.1186/s12870-022-03812-5.

## Background

As an important cereal and forage crop, maize plays an important role in sustaining global food security. Improvement of grain yield is a major and longstanding breeding goal for maize. Maize grain yield was determined by several yield-related traits, including grain yield per plant (GYP), ear length (EL), kernel row number (KRN), grain length (GL), grain width (GW), 100-kernel weight (HKW), and kernel number per row (KNR) [1]. Yield related traits possess higher heritability than grain yield and have great effects on improving grain yield [2]. They thus have attracted the attention of breeders in recent decades [3]. Nevertheless, our understanding of the molecular mechanisms underlying maize yield related traits is limited [4]. Identifying loci associated with yield related traits has become an essential topic in the molecular breeding practice of high yield maize which contributes to our understanding of the correlations between yield related traits at a molecular level.

Up to now, some yield related traits genes have been cloned by studying mutants [5–7]. Unfortunately, most of the traits related to plant development and yield in mutants show negative effects, which limits the application of mutants in breeding [8]. Therefore, the alleles controlling yield related traits can be identified by linkage mapping and association mapping in natural variation populations. To date, a number of quantitative trait loci (QTL) for yield related traits in maize have been detected by linkage analysis. Liu et al. [9] detected four QTL controlling KRN in an F_2_ population and two QTL controlling KRN in a recombinant inbred line (RIL) population derived from the crossing of the maize inbred lines abe2 and B73. Using an intermated B73 × Mo17 Syn10 doubled haploid population, Zhang et al. [10] detected 100 QTLs for yield related traits and eight significant SNPs co-located within intervals of seven QTLs. Through linkage analysis, a PPR family gene *ZmVPS29* regulating maize grain shape was successfully cloned according to genetic population which was constructed with maize inbred lines Huangzaosi and Lv28. Overexpression of *ZmVPS29* could make the grain slender and significantly improve the yield per ear of maize [11]. However, QTL with small effects were difficult to identify since classical biparental populations generally lead to relatively low resolution [12]. Furthermore, some rare alleles are often neglected due to the lack of genetic diversity in biparental populations [13].

As a cost-effective tool for dissecting the genetic architecture of complex quantitative traits, genome-wide association studies (GWAS) provide a high-resolution approach for the identification of QTL and have been widely used for the examination of QTL for yield-related traits in crops [14]. Huang et al. [15] used high-density SNP data and GWAS method to analyze 950 rice varieties in the world, and identified 10 trait loci related to yield in rice. To better understand the molecular mechanism underlying yield, Li et al. [16] investigated four yield-related traits of 133 soybean landraces by GWAS method and the results revealed five candidate genes associated with yield-related traits. Maize had high genetic diversity and contains many rare alleles in genome, which is very suitable to study the genetic basis of yield-related traits by GWAS [17, 18]. Using the association panel composed of 240 maize inbred lines and recombinant inbred lines, Zhang et al. [2] identified 23 QTLs and 25 significant SNPs related to HKW, KRN and KNR, including a stable locus (PKS2) related to KRN, HKW and kernel shapes. Zhang et al. [10] Used a natural population and B73 × Mo17 syn10 doubled hybridized haploid population, detected 100 QTLs and 138 SNPs of yield related traits, and found that 8 important SNPs were located in the interval of 7 QTLs. Luo et al. [19] used the GWAS method to identify a QTL-YIGE1, which regulates ear length by affecting pistillate floret number. Overexpression of YIGE1 can promote the growth of female inflorescence meristem (IM), thereby increasing panicle length and grain number per row, thus increasing yield. The GWAS method has been used for detecting loci that control yield related traits in maize, such as grain yield per plant (GYP) [20], ear length (EL) [21], kernel row number (KRN) [22], kernel length (KL) [23], kernel width (GW) [23], 100-kernel weight (HKW) [24], and kernel test weight (KTW) [10]. Therefore, the yield related traits of quantitative trait nucleotides (QTNs) can be effectively identified by GWAS method, and will improve our understanding of the molecular mechanism underlying kernel yield formation in maize.

Under the trend of increasing planting density and higher requirements for light energy utilization efficiency in modern breeding, the plant type of maize, such as tassel branch number (TBN), has a great correlation with the yield of maize [6]. At present, many genetic loci for the tassel branch number have been obtained by QTL mapping or GWAS analysis. Yi et al. [25] used F_2:3_ population with 266 families and RIL population with 301 families to locate QTLs for tassel length and tassel branch number, detected 15 and 16 QTLs respectively, of which 4 QTLs can be co-located by the two populations. Upadyayulia et al. [26] analyzed the tassel correlation traits of maize backcross population and detected 45 QTLs controlling the tassel correlation traits, of which the bnlg344-phi027 segment of bin9.02 can explain 14.6% of the phenotypic variation. The known *ramosa1* (*ra1*) gene controlling the development of tassel is located in bin7.02 within the QTL interval. Using US-NAM population and CN-NAM population, 63 QTLs controlling the tassel branch number and 62 QTLs controlling the length of tassel were identified by linkage analysis, and 965 QTNs significantly associated with the tassel branch number were detected by association analysis [27].

In the present study, we used an association panel of 291 maize inbred lines to identify the significant SNPs related to yield related traits by GWAS in different environments. The objective of the study was to map SNPs that are significantly associated with yield related traits and identify the candidate genes involved in yield related traits. Our results will improve the understanding of molecular mechanisms underlying maize yield related traits and provide novel molecular markers that may be used by breeders to develop superior varieties.

## Results

### Yield related tarit phenotypic variations

**Fig. 1:**
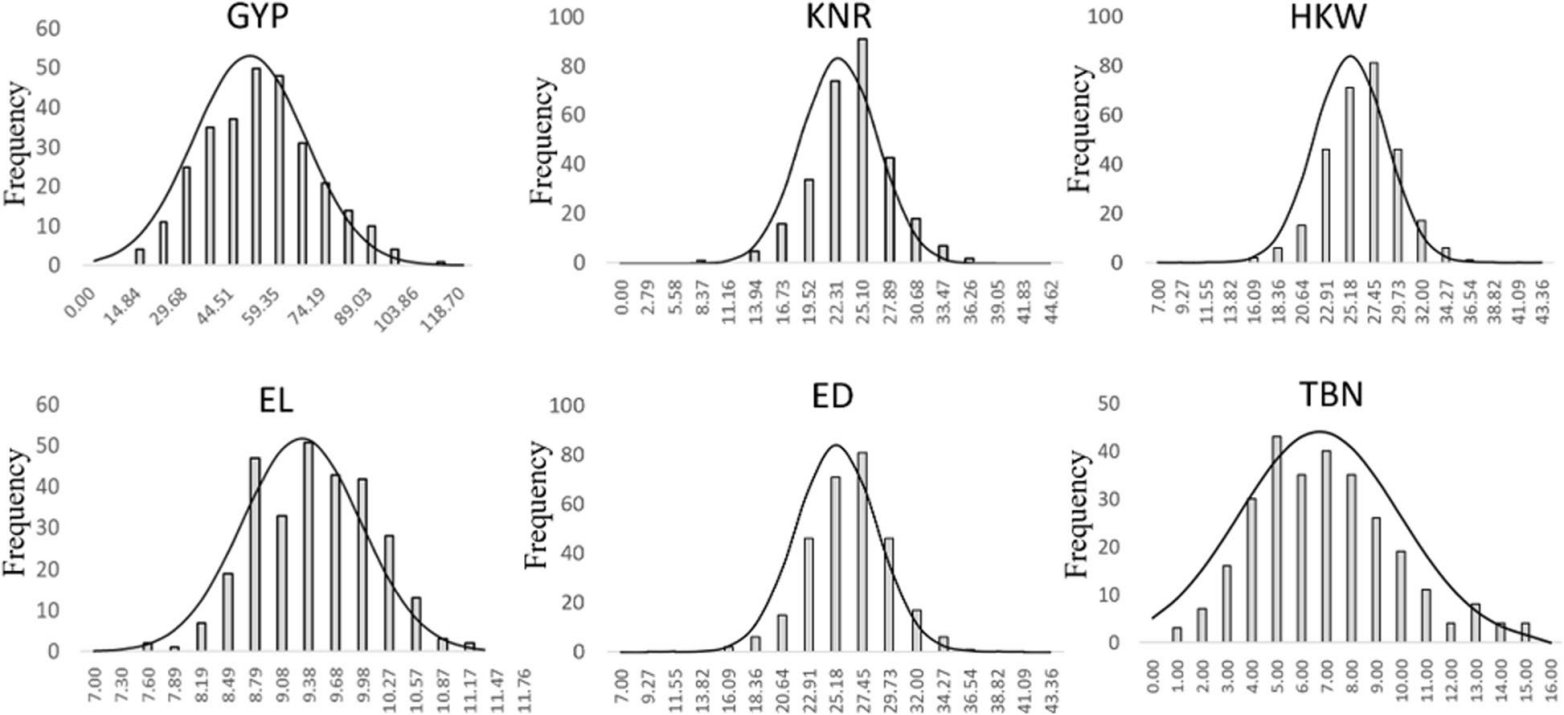
Phenotypic variation of yield related traits in 291 maize inbred lines

Taking the average value of yield related traits in 3 years as phenotypic data, the six yield related traits were statistically analyzed. The six phenotypic traits GYP, GW, GL, KNR, HKW and TBN of 291 maize inbred lines showed an approximate normal distribution (Fig. 1), indicating that these traits were typical quantitative traits controlled by multiple genes. Among 291 maize inbred lines, the phenotypes of GYP, KNR and TBN quantitative traits showed great variation (CV was 42.37, 39.95 and 49.79% respectively), and the 6 yield related traits showed high broad-sense heritability, which were 0.62, 0.65, 0.71, 0.61, 0.76 and 0.83 respectively (Table 1).

**Table 1:** Statistical analysis of yield related traits

Trait	Mean	Max	Min	SD	CV (%)	*H*^*2*^
GYP	49.92	127.50	3.39	21.15	42.37%	0.62
GW	8.34	30.60	4.97	0.96	11.51%	0.65
GL	9.32	12.47	6.10	1.07	11.43%	0.71
KNR	26.46	53.50	5.33	10.57	39.95%	0.61
HKW	25.18	40.04	9.75	4.93	19.59%	0.76
TBN	6.70	21.00	1.00	3.34	49.79%	0.83

*GYP* grain yield per plant (g), *GW* grain width (cm), *GL* grain length (cm), *KNR* kernel number per row, *HKW* 100-kernel weight (g), *TBN* tassel branch number. The same as below

### Group structure analysis of association penal

**Fig. 2:**
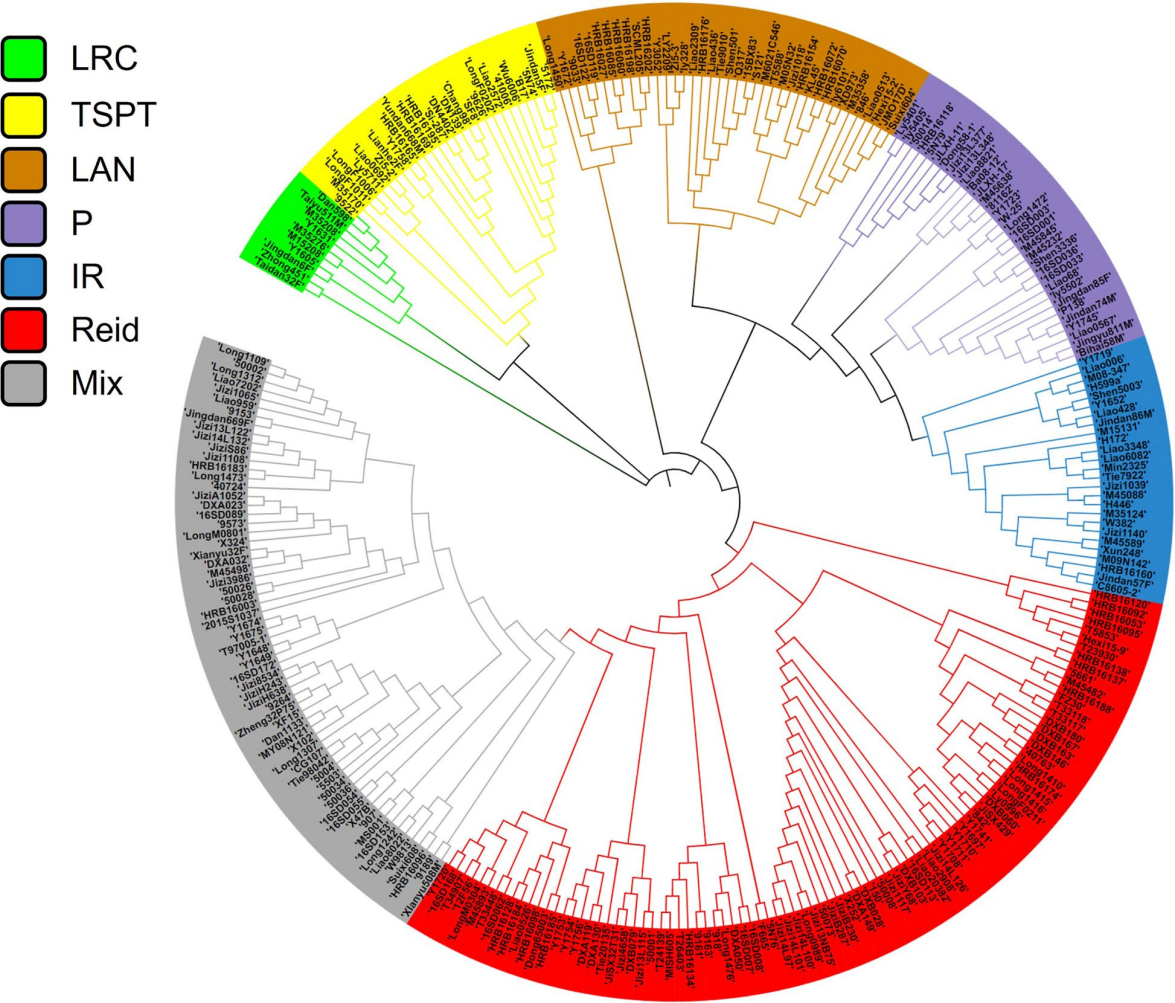
Cluster analysis of 291 maize inbred lines

Based on the genotypes of 291 inbred lines, according to TASSEL5.0 software for cluster analysis, combined with the analysis results of Li et al. [28] on the population structure, 291 materials were clustered (Fig. 2). When 50% group attribute ratio was used as the basis for classification, 227 (78.0%) of 291 inbred lines were divided into 6 groups: Lüda red cob group (LRC), Tangsipingtou group (TSPT), Lancaster group (LAN), P group (P), Improved Reid group (IR) and Reid group (Reid); while the remaining 64 lines did not have clear group attribution characteristics and were classified as mixed groups (Mix). Among the seven groups, Lüda red cob group, Tangsipingtou group, Lancaster group, P group, Improved Reid group and Reid group, contain 10, 27, 39, 33, 26 and 92, materials respectively, accounting for 3.4, 9.3, 13.4, 11.3, 8.9 and 31.6% of the total materials respectively. Lüda red cob group, Tangsipingtou group, Lancaster group, P group, Improved Reid group and Reid group have been reported in previous studies, and they are all commonly used heterosis groups in maize breeding [29, 30]. The materials in the mixed population contained Chinese and foreign germplasm widely, so the association panel had a wide genetic basis and rich yield related variation loci.

### Genome wide association analysis of yield related traits

**Fig. 3:**
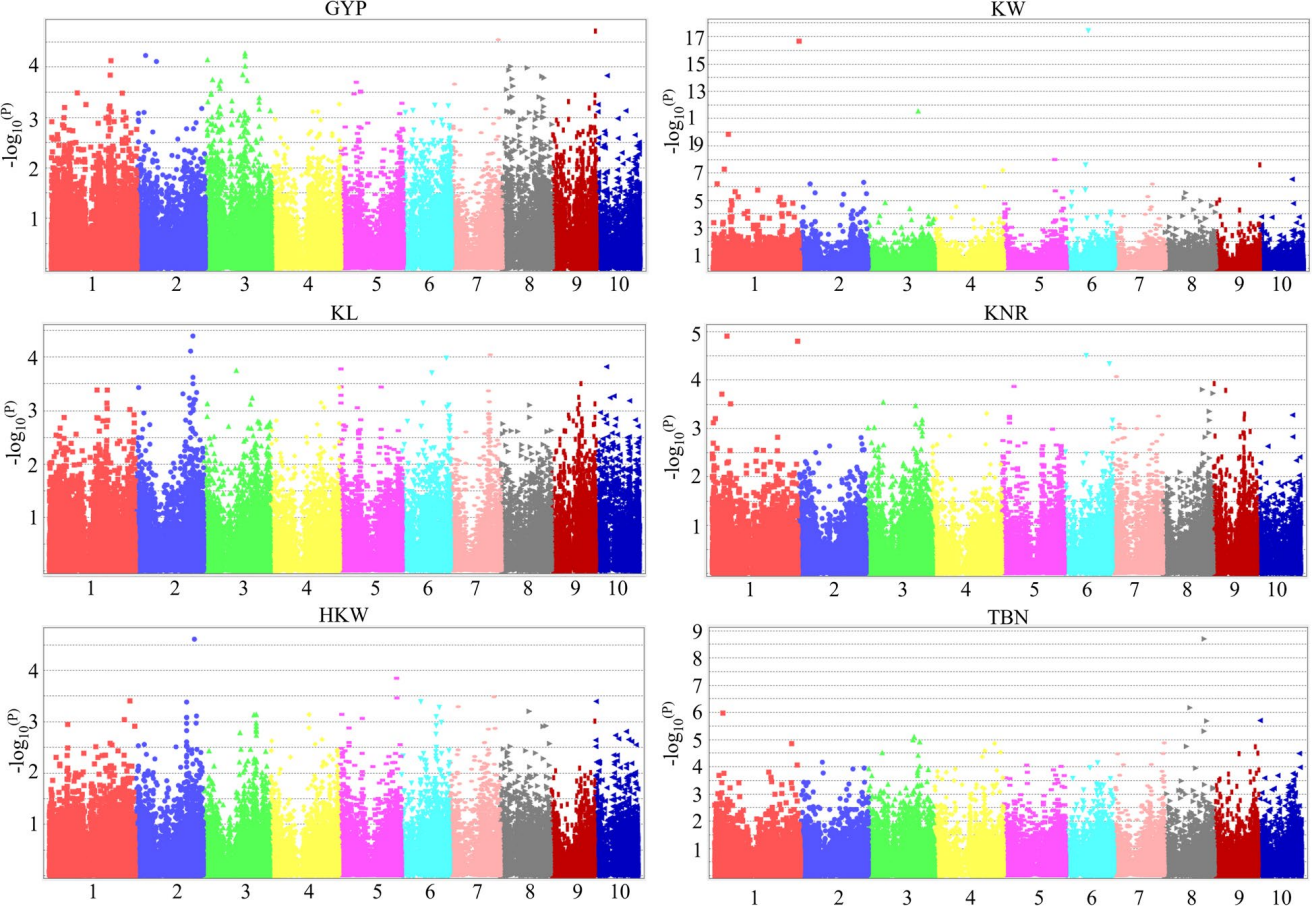
Manhattan plot for genome-wide association study of maize yield related traits

In total, 38,683 high-quality SNPs were used to perform GWAS for six yield related traits. MLM with PCA model was used to analyze the average values of yield related traits of 291 maize inbred lines in 6 environments. The GWAS results showed that a total of 59 significantly associated yield related SNPs were identified, and their *p* values were less than 0.0001 or could be detected in two yield related traits (Fig. 3 and Table 2). Among the significantly SNPs, 11 SNPs of GYP were detected, which were located on chromosomes 1, 2, 3, 7, 8, 9 and 10; 29 SNPs of GW were detected, which were located on all chromosomes; 4 SNPs of GL were detected, which were located on chromosomes 2, 7 and 10; 5 SNPs of KNR were detected, which were located on chromosomes 1, 6 and 7; 2 SNPs of HKW were detected, which were located on chromosomes 2 and 6; 11 SNPs of TBN were detected, which were located on chromosomes 1, 3, 4, 7, 8 and 10. At the same time, three of these SNPs can be detected in two different traits (bold SNPs, Table 2).

**Table 2:** List of significant SNPs associated maize yield related traits and the candidate genes and their functional annotations

Trait	SNP	*P* value	R^2^%	Candidate gene	Gene annotation
GYP	9_150257246	1.92E-05	7.77	GRMZM2G330945	Cold regulated gene 27 (COR27)
GYP	7_162001602	2.86E-05	8.50	GRMZM2G151649	ARM repeat superfamily protein
GYP	3_138419644	5.32E-05	7.01	GRMZM5G803355	MYB transcription factor
GYP	2_29336901	5.90E-05	6.94	GRMZM5G845736	Inactive beta-glucosidase
GYP	3_138419203	6.10E-05	6.91	GRMZM5G803355 GRMZM2G585025	MYB transcription factor Small RNA degrading nuclease 5
GYP	3_7417157	7.15E-05	6.79	GRMZM2G015267	FAD/NAD(P)-binding oxidoreductase
GYP	1_209009744	7.45E-05	6.76	GRMZM2G125557	Auxin-repressed protein putative expressed
GYP	2_66831336	7.76E-05	6.73	GRMZM2G097406	Unknown
GYP	3_138762178	9.58E-05	6.58	GRMZM2G087590	PsbP domain-containing protein 4
GYP	8_29023337	9.72E-05	6.57	GRMZM2G393347	HOPZ-ACTIVATED RESISTANCE 1 (ZAR1)
GYP	**10_34938698**	1.48E-04	6.26	**GRMZM2G003090**	Unknown
KW	**6_77081642**	3.60E-18	32.43	**GRMZM2G328197** **GRMZM2G376957**	RING zinc finger domain superfamily protein Histone H3-like 5
KW	**1_299177196**	2.17E-17	32.56	**GRMZM2G110851**	Pentatricopeptide repeat-containing protein
KW	3_175569291	2.86E-12	20.42	GRMZM2G149662	COV1-like protein
KW	1_52,668,969	1.42E-10	18.58	GRMZM2G174696	Mitochondrial import receptor subunit TOM40–1
KW	5_177277411	1.02E-08	15.00	GRMZM2G492156	MADS-box transcription factor 27
KW	9_154673101	2.50E-08	14.28	GRMZM2G092741	ARATH AP-2 complex subunit alpha-2
KW	6_67479669	2.53E-08	13.00	GRMZM2G430902	C3HC4-type RING finger family protein
KW	1_38858313	5.16E-08	12.44	AC204035.3	Unknown
KW	4_238037247	6.29E-08	12.28	GRMZM2G166145	Apoptosis-inducing factor homolog
KW	10_110533455	2.82E-07	12.31	GRMZM2G153215	Membrane-anchored ubiquitin-fold protein 4
KW	2_223591728	4.83E-07	10.70	GRMZM2G172101 GRMZM2G052507	Seryl-trna synthetase Serine carboxypeptidase-like 45
KW	2_36952454	6.26E-07	11.67	GRMZM2G102238	PAP2 family domain containing protein
KW	1_13682335	6.28E-07	10.49	GRMZM2G417455	Beta-galactosidase 5
KW	7_130909742	6.31E-07	10.49	GRMZM2G464985	Serine/threonine-protein kinase D6PKL1
KW	4_174067645	9.84E-07	10.15	GRMZM2G010933	Cytochrome c oxidase copper chaperone 1
KW	6_66363963	1.72E-06	9.72	GRMZM2G110983	Ubiquitin-conjugating enzyme E2
KW	1_154806752	1.76E-06	9.70	GRMZM2G446047	Trm32_arath protein trm32
KW	5_179440519	2.05E-06	9.58	GRMZM2G003384	60S ribosomal protein L6
KW	1_76348752	2.30E-06	10.62	GRMZM2G174708	Polygalacturonase inhibitor 1
KW	6_19391683	2.56E-06	10.54	GRMZM2G319397	Unknown
KW	8_71170430	2.78E-06	10.47	GRMZM5G887975	GATA transcription factor 19
KW	2_54135317	2.80E-06	9.35	GRMZM2G173218	Unknown
KW	2_233033601	3.21E-06	9.24	GRMZM5G843555 GRMZM2G149935	Putative prolyl 4-hydroxylase 12 Hydroxyproline o-galactosyltransferase galt4
KW	2_153726247	3.51E-06	9.17	GRMZM5G800842	Ubiquitin-activating enzyme E12
KW	7_115163058	5.27E-06	8.86	GRMZM2G071059 GRMZM2G171408	CCR4-NOT transcription complex subunit 7 Spotted leaf protein 11
KW	1_87304388	5.56E-06	8.82	GRMZM2G046848	U-box domain-containing protein kinase family
KW	1_233620848	6.16E-06	8.74	GRMZM2G001850	Nucleolin like 2
KW	5_204816347	6.48E-06	8.70	GRMZM5G899787	RNA-binding (RRM/RBD/RNP motifs) protein
KW	8_65840306	6.66E-06	8.68	GRMZM2G477340	CDP-diacylglycerol--serine O-phosphatidyltransferase 2
KL	2_200248530	4.01E-05	7.18	GRMZM2G435689	Unknown
KL	2_192310952	7.72E-05	6.70	GRMZM2G013892	Zinc finger C3HC4 type domain containing protein
KL	7_137256260	9.02E-05	6.59	GRMZM2G458164	Glucan endo-1 3-beta-glucosidase precursor
KL	**10_34938698**	1.51E-04	6.21	**GRMZM2G003090**	Unknown
KNR	1_52253410	1.23E-05	9.29	GRMZM2G128644	VQ motif-containing protein
KNR	**1_299177196**	1.58E-05	9.09	**GRMZM2G110851**	PPR repeat domain containing protein
KNR	**6_77081642**	3.10E-05	7.52	**GRMZM2G328197** **GRMZM2G376957**	RING zinc finger domain superfamily protein Histone H3-like 5
KNR	6_158099344	4.58E-05	8.25	GRMZM2G049091 GRMZM2G138067	Transcription initiation factor IIF beta subunit Protein LURP-one-related 5
KNR	7_13586175	8.49E-05	7.77	GRMZM2G446921	F-box domain containing protein
HKW	2_206427709	2.45E-05	8.72	GRMZM2G418343 GRMZM2G117900	Cell wall protein precursor putative Translation initiation factor family protein
HKW	6_67617018	4.05E-04	5.59	GRMZM2G430902	C3HC4-type RING finger family protein
TBN	8_139,471,588	2.00E-09	14.76	GRMZM2G101664	Zinc finger protein
TBN	8_89433292	6.73E-07	10.25	GRMZM5G856067 GRMZM2G063676	Ribosomal protein L18ae family heat shock 70 kDa protein 4
TBN	1_29024922	1.07E-06	9.90	GRMZM2G153611 GRMZM2G180023	E3 ubiquitin-protein ligase ARI2 serine/threonine receptor-like kinase NFP
TBN	10_4247900	1.98E-06	9.44	GRMZM5G886547	Non-specific lipid-transfer protein 3
TBN	8_148198954	2.07E-06	9.40	GRMZM2G130586	Unknown
TBN	8_139164894	4.92E-06	8.76	GRMZM2G046037	ubiquitin carboxyl-terminal hydrolase MINDY-2
TBN	3_160368430	7.87E-06	8.40	GRMZM2G129114	Nucleotide-diphospho-sugar transferase
TBN	1_270000781	1.41E-05	7.97	GRMZM5G857351	translational activator GCN1
TBN	3_180017439	1.20E-05	8.09	GRMZM2G042295	methyltransferase putative expressed
TBN	4_209597837	1.39E-05	7.98	GRMZM2G094541	Receptor-like serine/threonine-protein kinase SD1–6
TBN	7_175448419	1.32E-05	8.02	GRMZM2G086628	early nodulin-like protein 16

Bold represents the SNPs associated with two yield related traits

### Candidate genes involved in yield related traits

The LD analysis results of this association panel showed that when *r*^2^ > 0.2, the LD decay with physical distance in our association panel was calculated to be 100 kb (Fig. S1). SNPs with significant correlation were screened out from GWAS. The yield related candidate genes within the LD range of significant association sites were found on the maizeGDB website (B73 RefGen_v4). A total of 66 candidate genes were identified in 59 SNPs controlling yield related traits, of which 58 had functional annotation (Table 2).

## Discussion

### Abundant phenotypic variations in the yield related traits

At present, GWAS method has been widely used to study the genetic basis of important traits of many species by calculating the association between genotypic and corresponding phenotypic variations [31]. In the study conducted by Zhang et al. [10], the population had a large phenotypic variation in ERN (ear row number), ranging from 9.00 to 20.10; in HKW, ranging from 14.84 to 41.75 g; in KNR, ranging from 14.50–35.05; in EGW (ear grain weight), ranging from 102.70–801.75 g. Meanwhile, in the study of Ma et al. [20], phenotypic variation of the association panel in the BLUE (best linear unbiased estimate) value of GYP was 42.2 g, CV 40%; the BLUE value of HKW is 26 g, CV 17%; the BLUE value of KNR was 15.87, CV 28%. Greater phenotypic variation would be beneficial for dissecting the genetic architecture of the yield related traits. Among the association panel composed of 291 inbred lines had a large phenotypic variation in GYP, GW, GL, KNR, HKW and TBN (Table 1), so the association panel was suitable for association analysis of yield related traits.

### Genetic architecture of yield related traits

Crop yield is a complex quantitative trait. Understanding the genetic structure of maize yield is helpful to maize high-yield breeding. GWAS facilitates the identification of QTNs and candidate genes associated with the target traits. In this study, we performed GWAS using the association panels, including 291 inbred lines with 38,683 SNP markers, we obtained 59 significant SNPs (*P* < 0.0001) that were significantly associated with six yield related traits in maize. Among these SNPs, some overlapped with previously reported QTL/QTN intervals. The SNP 9_150257246 (Chr9: 150.25 Mb), 7_162001602 (Chr7: 162.00 Mb) and 1_209009744 (Chr1: 209.00 Mb) of GYP were mapped to the previously detected QTL Yqgypp9 (Chr9: 140.8–158.6 Mb), qgy-7.2 (Chr9: 161.51–165.72 Mb), the QTL detected in RIL population derived from lines DAN340 × K22 (Chr1: 208.36–209.3 Mb) [32–34]. the GYP-associated SNP 7_162001602 (Chr7: 162.00 Mb) was closely located with the SNP chr7.S_162987283 (Chr7:162.98 Mb) detected in the RIL population [34]. Four GW-related SNPs 2_36952454 (Chr2:36.95 Mb), 2_54135317 (Chr2:54.13 Mb), 3_175569291 (Chr3:175.56 Mb) and 5_177277411 (Chr5:177.27 Mb) were mapped to the previously reported intervals of the GW-related QTL on Chr2: 33.71–36.47 Mb, Chr2: 45.2–54.97 Mb, Chr3: 175.56–179.42 Mb and Chr5: 168.68–177.86 Mb [21, 34, 35]. 7_137256260 (Chr7: 137.25 Mb) that was associated with GL situated closely the interval of the GL SNP chr7.S_137701632 (Chr7: 137.70 Mb) identified in the RIL population [34]. The SNP 8_139,471,588, 8_139164894 and 8_148198954 of TBN were located closely and mapped to the previously detected QTL of TBN in qTBN8–1 (Chr8: 129.97–154.67 Mb) [22]. SNP 8_89433292 (chr8: 89.43 Mb) associated with TBN and located in the QTL interval of Q49_CN-NAM_ (chr8: 73-101 Mb), which was positioned by Wu et al. [27] in TBN. The SNP 3_180017439 (chr3: 180.01 Mb) of TBN was closely linked to the SNP S3_179732428 (chr3: 179.73 Mb) and 179,982,665 (chr3: 179.98 Mb) of TBN [4, 27]. These yield related SNPs could be considered population-stable SNPs, which should be given close attention in MAS breeding for improving maize yield. In addition, several SNPs not found in previous studies might contribute to achieving high and stable yield in maize.

### Pleiotropic loci affect yield related traits in maize

Pleiotropism is a common phenomenon that has been found in the QTL mapping and GWAS of multiple crops [36, 37]. According to combined linkage and association mapping, Zhang et al. [2] found 17 QTL/SNPs which had pleiotropism in yield related traits in maize. Liu et al. [23] investigated in an association panel and a biparental population, and also identified five pleiotropic QTLs for kernel traits, which implicating that a close genetic correlation existed among different kernel traits in maize. In our study, we identified 3 pleiotropic SNPs (pSNP) that have pleiotropic effect on different yield related traits (Table 2 bold SNP). Of these, pSNP 1_299177196 and 6_77081642 displayed a pleiotropic effect on GW and KNR. The SNP 1_299177196 associated candidate gene was GRMZM2G110851, which encoded a pentatricopeptide repeat-containing (PPR) protein. Chen et al. [11] cloned the PPR family gene *Zmvps29* through linkage analysis, which can regulate the kernel width of maize and increase the kernel number per row. GRMZM2G110851 and *Zmvps29* both belong to PPR family genes and have the same regulatory effect on maize kernel, suggesting that GRMZM2G110851 has a similar function with *Zmvps29*. The candidate gene GRMZM2G328197 of SNP 6_77081642 in GW and KNR encoded a RING zinc finger domain superfamily protein, which was previously reported to be significantly related to panicle length in rice and to have a positive role in seed germination in *Arabidopsis* [37, 38]. The pSNP 10_34938698 could be detected in both GYP and GL which associated with a candidate gene GRMZM2G003090, but its function was unknown. These pleiotropic SNPs detected in multiple yield related traits might be stable sites for regulating maize yield, which was helpful to understand the molecular mechanism of maize yield formation.

### Candidate genes involved in yield related traits

Among these candidate genes in this study, some of them were previously reported to affect grain yield or kernel development, which were considered the top-priority candidate genes. The SNP 3_138419644 and 3_138419203 were both associated with GRMZM5G803355, which encoded an MYB transcription factor. Jia et al. [39] found that the expression of *ZmMYBE1* in the two hybrids was higher than that in their parents, and considered that *ZmMYBE1* was related to yield heterosis at the transcriptional level. The candidate gene of SNP 6_67617018 and 6_67479669 were GRMZM2G430902, which encoded a C3HC4 type ring finger family protein. The family genes were expressed in many tissues of Arabidopsis and maize during reproductive development, also played an important role in plant seed development [40]. The SNP 1_ 52,668,969 had a high *P*-value, which associated gene GRMZM2G174696 encoded a TOM40 protein. TOM40 was relatively conservative and had homologous genes in rice and maize. In *Arabidopsis*, *AtTOM40* was essential for the normal structure of the mitochondrion, and participated in early embryo development and pattern formation through maintaining the biogenesis of mitochondria [41]. The candidate gene GRMZM2G304745 of SNP 2_23576028 encoded a leucine-rich repeat protein kinase family protein, overexpression of *LRK* (leucine-rich repeat receptor kinase) gene could increase the yield of rice [42]. GRMZM5G878070 encoded a ABC1-like kinase protein, overexpressing *OsAGSW1* (ABC1-like kinase related to Grain size and Weight) exhibited a phenotype with a significant increase in grain size, grain weight, grain filling rate and 1000-grain weight compared with the wild-type and RNAi transgenic plants in rice [43]. GRMZM2G492156 encoded a MADS-box transcription factor 27 protein, overexpressing MADS-box showed new attributes such as the increase of vegetative growth and grain weight in maize [44]. GRMZM2G464985 annotated as a serine/threonine-protein kinase gene, was previously demonstrated to play vital roles in ear length, kernel number and enhance maize hybrids grain yield [45]. The SNP 8_ 139,471,588 had the most significant *p*-value in the TBN, which can explain 14.76% of the phenotypic variation of TBN. This locus was associated with GRMZM2G101664, which encoded a zinc finger protein. *NSG1* encoded a member of the zinc finger protein family and was expressed mainly in the organ primordia of the spikelet in rice, which played a pivotal role in maintaining organ identities in the spikelet by repressing the expression of LHS1, DL, and MFO1 [46]. Maize *ramosa1* (*ra1*) gene encoded a zinc finger transcription factor protein, which was involved in the regulation of tassel development in maize [47]. The zinc finger protein encoded by GRMZM2G101664 might also be involved in the development of maize tassel. The SNP 8_89433292 could explain 10.25% of the phenotypic variation and associated with GRMZM2G042295, which encoded a heat shock protein. HSP101 can participate in the regulation of tassel development at the post transcriptional level in maize [48]. The SNP 3_180017439 associated with GRMZM2G042295, which encoded a methyltransferase family protein. Wang et al. [49] found a methyltransferase family protein and played a key role in the regulation of secondary wall biosynthesis in interfascicular fibers during inflorescence stem development of Arabidopsis. These genes are considered to be reliable candidate genes for regulating yield related traits in maize, and further verification of their function will be helpful for further elucidating the underlying genetic and molecular mechanisms of yield related traits.

## Conclusion

In this study, a genome-wide association study (GWAS) method was made on an association panel of 291 inbred lines. Using 38,683 high-quality SNPs, six yield related traits were analyzed by the MLM with PCA method. A total of 59 yield related SNP were detected, involving 66 candidate genes. In the future, it is expected to improve the accuracy of GWAS results by adding more representative inbred lines to expand the association population and identifying high-quality phenotypic data from multiple environmental trials. Our results will improve the understanding of the genetic and molecular mechanisms underlying maize grain yield as well as provide new molecular markers for breeders to develop superior maize varieties.

## Method

### Plant material and field experiments

An association panel of 291 wide range of genetic diversity maize inbred lines in China, was collected for GWASs. All the accessions were planted following a randomized block design of two replicates in 3 years (2017, 2018, 2019) Jinan in Shandong Province (E117°10′, N36°25′). Each material was planted in a row. The field experiments include in a single row 3 m in length, with 0.6 m between adjacent rows and 14 individual plants per row. The Maize Institute of Shandong Academy of Agricultural Sciences has established experimental field bases at Jinan. The field experiments were approved by the Maize Institute, and field management followed local maize management practices. The field studies did not involve endangered or protected species in this study. We declare that all plant materials comply with the ‘Convention on the Trade in Endangered Species of Wild Fauna and Flora’ in this study. The plant materials used in this study were conserved in our lab.

### Phenotyping and data analysis

The phenotypic traits measured in this study included grain yield per plant (GYP), grain length (GL), grain width (GW), kernel number per row (KNR), 100-kernel weight (HKW) and tassel branch number (TBN). In GYP, the ears of each line were harvested after reaching maturity and 10 ears with consistent growth were selected for evaluation in each replication. In GL, GW and KNR, the phenotypes were represented by the mean values of 10 ears. In HKW, the average weight of three repeated measures of 100 randomly selected kernels from the bulked kernels of each line. TBN was the average number of tassel branch number of 10 random individual plants in each row.

Excel 2016 and SPSS16 software were used to make statistical analysis on six traits, including GYP, GL, GW, HKW, KNR and TBN. The average values of each trait of 3 years were taken, and the standard deviation and coefficient of variation of each trait were calculated (Table S2). QTL IciMapping V4.1 was used to calculate broad-sense heritability (*H*^*2*^) by ANOVA in software [50]. The coefficient of variation was calculated as CV(Coefficient of variation) = SD(Standard Deviation)/Mean [28].

### DNA extraction and genotyping

Five maize plants were selected from each material, and their fresh leaves were used to extract genomic DNA. We extracted the genomic DNA followed the cetyltrimethylammonium bromide (CTAB) method [51]. All samples were quality checked and genotyped using the GenoBaits Maize 40 K chip [52]. Then, the successfully called SNPs with a missing rate of more than 20% and minor allele frequency (MAF) of < 0.05 were excluded from the genotyping dataset [53]. After that, 38,682 high-quality SNPs were used in further analysis (Table S1).

### Genome-wide association studies

All the above phenotypic and genotypic data in the above associated population were used for GWAS. Based on high-quality SNPs, TASSEL 5.0 software was used to analyze the population structure of 291 inbred lines. Combined with the material pedigree, iTOL software was used to draw neighbor joining tree [54]. Using MLM with principal components analysis (PCA) model by TASSEL 5.0, we carried out GWAS for the six yield related traits investigated in this study [55]. The suggestive *P* value (0.05/N) was set as a significance threshold and N was calculated using the simpleM package in R to control false negatives [56].

### Candidate genes identification

We examined the LD in the genomic region around each significant SNP to establish a supporting interval for the significant association. That supporting interval would comprise the surrounding region in LD (*r*^2^ > 0.2) [57]. The candidate genes in the LD region around significant SNPs were identified based on the B73 reference genome V3 from MaizeGDB (https://www.maizegdb.org/).

## Supplementary Information

**Additional file 1.**

**Additional file 2.**

**Additional file 3.**

**Additional file 4.**

## Data Availability

The sequencing data generated in this study were deposited in the Sequence Read Archive (SRA) databases (PRJNA842838). In addition, we have also sorted out a genotype data Table for GWAS based on the sequencing data, please refer to Table S1 for details.

## Abbreviations

GYP: yield per plant
GL: grain length
GW: grain width
KNR: kernel number per row
HKW: 100 kernel weight
TBN: tassel branch number
EL: ear length
KRN: kernel row number
KL: kernel length
ERN: ear row number
EGW: ear grain weight
GWAS: Genome-wide association study
LD: Linkage disequilibrium
MLM: Mixed linear model
QTL: Quantitative trait locus
QTN: Quantitative trait nucleotide
SNP: Single nucleotide polymorphism
