# Supplementary figures and images for: Genome wide association analysis for yield related traits in maize
Source: BMC Plant Biol. 2022 Sep 21;22:449. doi: 10.1186/s12870-022-03812-5 (PMC9490995; doi:10.1186/s12870-022-03812-5)

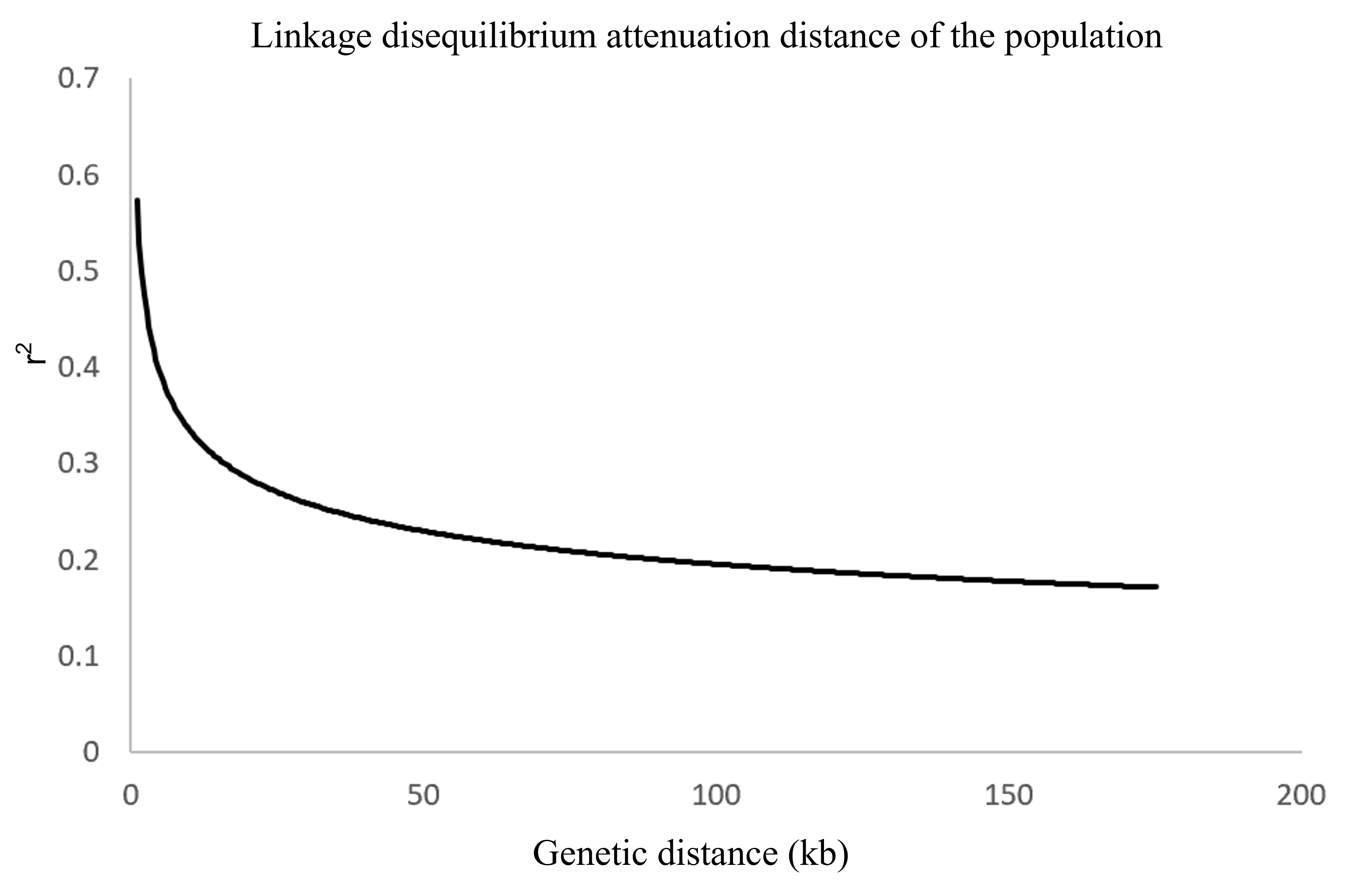

Supplement: Supplementary file 1 — Additional file 1. [file 12870_2022_3812_MOESM1_ESM.tif]
