# Supplementary material for: Genome wide association analysis for yield related traits in maize
Source: BMC Plant Biol. 2022 Sep 21;22:449. doi: 10.1186/s12870-022-03812-5 (PMC9490995; doi:10.1186/s12870-022-03812-5)
Supplement: Supplementary file 2 — Additional file 2. [file 12870_2022_3812_MOESM2_ESM.docx]

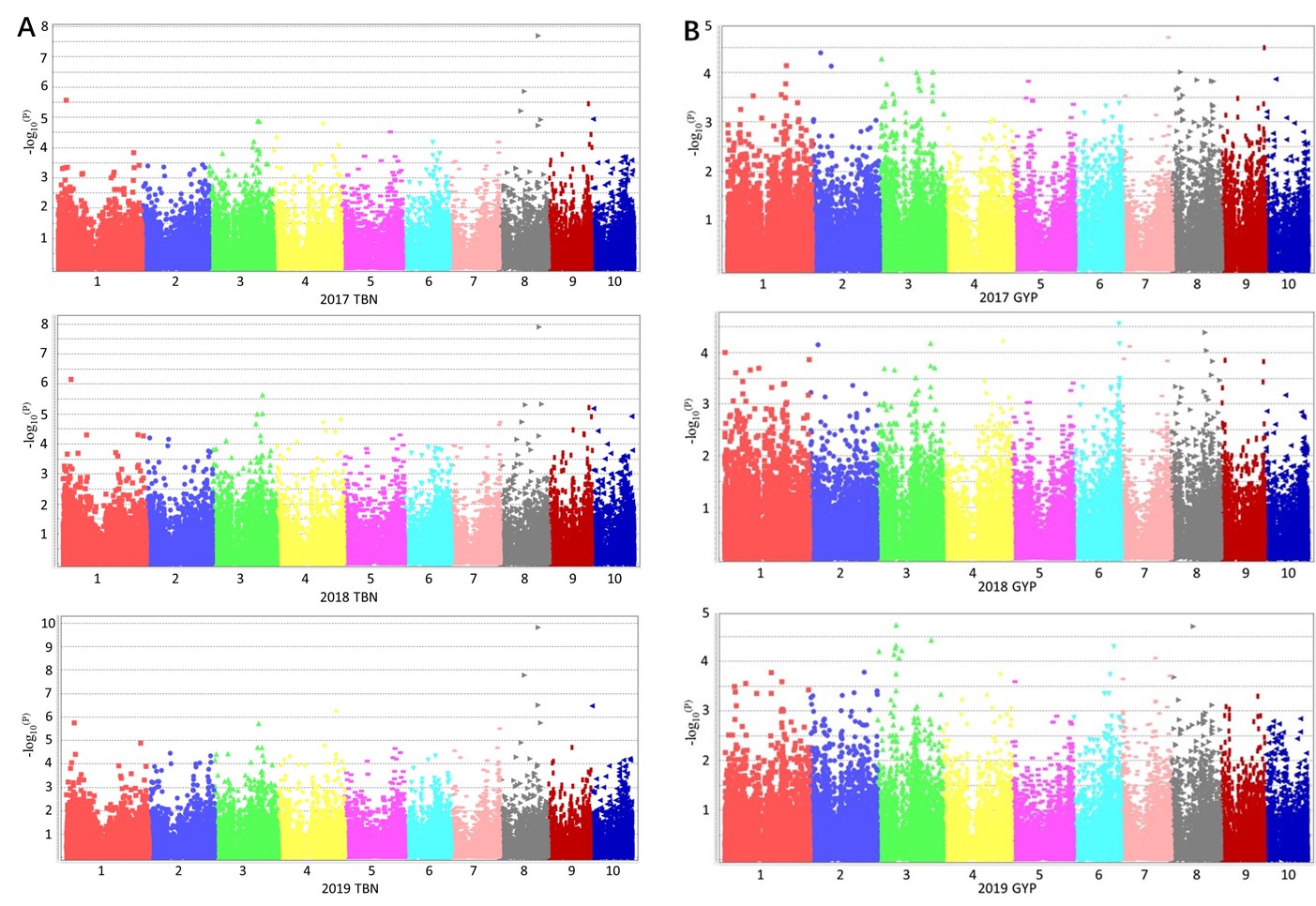


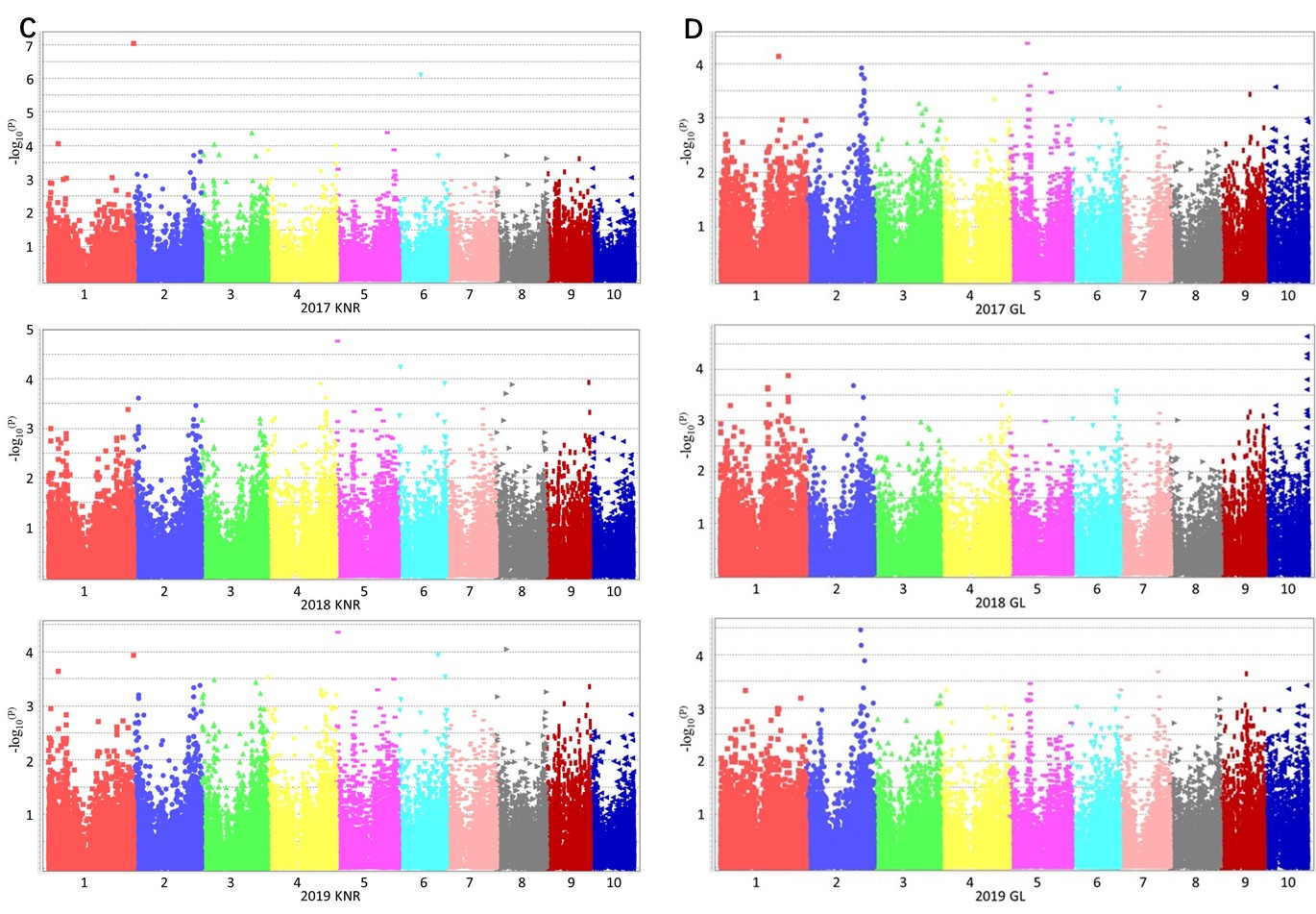


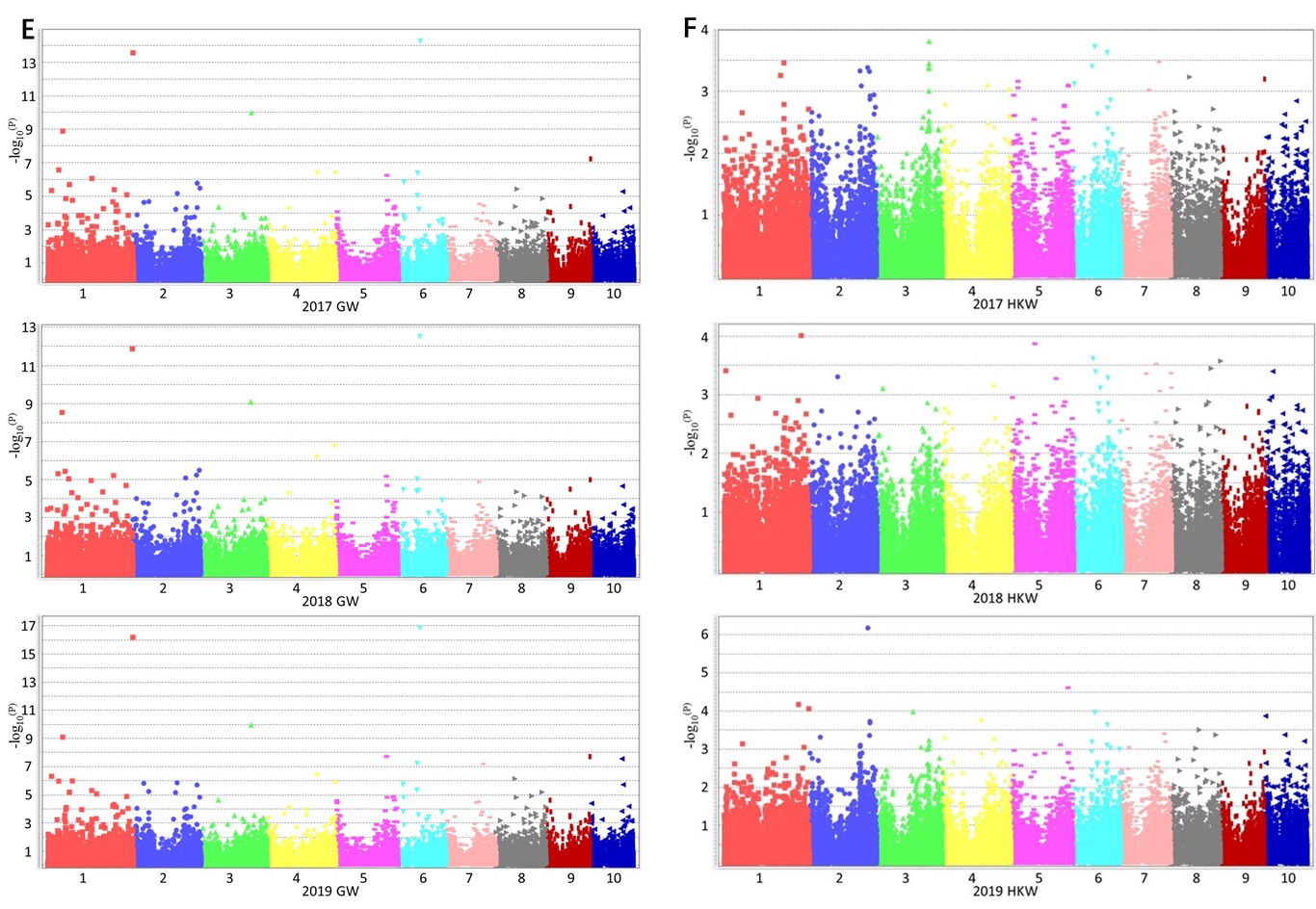


Fig S2 Manhattan plot for genome-wide association study of maize yield related traits in 2017, 2018 and 2019. GYP: grain yield per plant (g); GW: grain width (cm); GL: grain length (cm); KNR: kernel number per row; HKW: 100-kernel weight (g); TBN: tassel branch number.
